# Routine laboratory biomarkers used to predict Gram-positive or Gram-negative bacteria involved in bloodstream infections

**DOI:** 10.1038/s41598-022-19643-1

**Published:** 2022-09-14

**Authors:** Daniela Dambroso-Altafini, Thatiany C. Menegucci, Bruno B. Costa, Rafael R. B. Moreira, Sheila A. B. Nishiyama, Josmar Mazucheli, Maria C. B. Tognim

**Affiliations:** 1 Laboratório de Microbiologia, Department of Basic Health Sciences, State University of Maringá, Avenida Colombo 5790, Maringá, Paraná 87020-900 Brazil; 2 Department of Medicine, University Paranaense, Umuarama, Paraná Brazil; 3 Maringá University Hospital, State University of Maringá, Maringá, Paraná Brazil; 4 Department of Statistic, State University of Maringá, Maringá, Paraná Brazil

**Keywords:** Microbiology, Biomarkers

## Abstract

This study evaluated routine laboratory biomarkers (RLB) to predict the infectious bacterial group, Gram-positive (GP) or Gram-negative (GN) associated with bloodstream infection (BSI) before the result of blood culture (BC). A total of 13,574 BC of 6787 patients (217 BSI-GP and 238 BSI-GN) and 68 different RLB from these were analyzed. The logistic regression model was built considering BSI-GP or BSI-GN as response variable and RLB as covariates. After four filters applied total of 320 patients and 16 RLB remained in the Complete-Model-CM, and 4 RLB in the Reduced-Model-RM (RLB p > 0.05 excluded). In the RM, only platelets, creatinine, mean corpuscular hemoglobin and erythrocytes were used. The reproductivity of both models were applied to a test bank of 2019. The new model presented values to predict BSI-GN of the area under the curve (AUC) of 0.72 and 0.69 for CM and RM, respectively; with sensitivity of 0.62 and 0.61 (CM and RM) and specificity of 0.67 for both. These data confirm the discriminatory capacity of the new models for BSI-GN (p = 0.64). AUC of 0.69 using only 4 RLB, associated with the patient's clinical data could be useful for better targeted antimicrobial therapy in BSI.

## Introduction

Correct and immediate antimicrobial treatment significantly reduces the mortality of patients with bloodstream infections (BSI), which affects about 30 million people, and are among the main causes of morbidity, causing approximately 6 million deaths per year worldwide^1–4^. Although the confirmation of BSI only occurs definitively with positive blood culture (BC), the complete identification and antimicrobial process of susceptibility testing of the etiologic agent takes time (48–72 h), which delays the correct choice of treatment^5–7^. Thus, faster and more practical alternatives that can predict the bacterial group, whether Gram-positive (GP) or Gram-negative (GN), responsible for BSI, could be extremely important to target antibacterial therapy.

In order to predict bacteremia, mortality and sepsis, several authors have proposed the use of laboratory biomarkers^1,5,6,8–10^. However, few studies have been conducted to search for other biomarkers that may be related to the bacterial group involved in BSI.

Ratzinguer et al. evaluated a large number of biomarkers with statistical analysis and did not obtain a satisfactory result^11^. However, Lin et al. and Li et al. demonstrated that some biomarkers, when associated, had the potential to predict the bacterial group involved in BSI^12,13^. Recently, Tang et al. pointed out that specific combinations of hematological parameters can prove its power to distinguish patients with BSI caused by different pathogens, including GP and GN bacteria^14^.

Considering the great importance of the topic and the complex and controversial results, new studies and research need to be reported to better understand the role of routine biomarkers used in laboratories in predicting the bacterial group involved in BSI. The present study aimed to develop a model that predicts whether a bloodstream infection is caused by a GN or a GP organism using routine laboratory biomarkers (RLB).

## Results

### Study design and data collection

**Figure 1:**
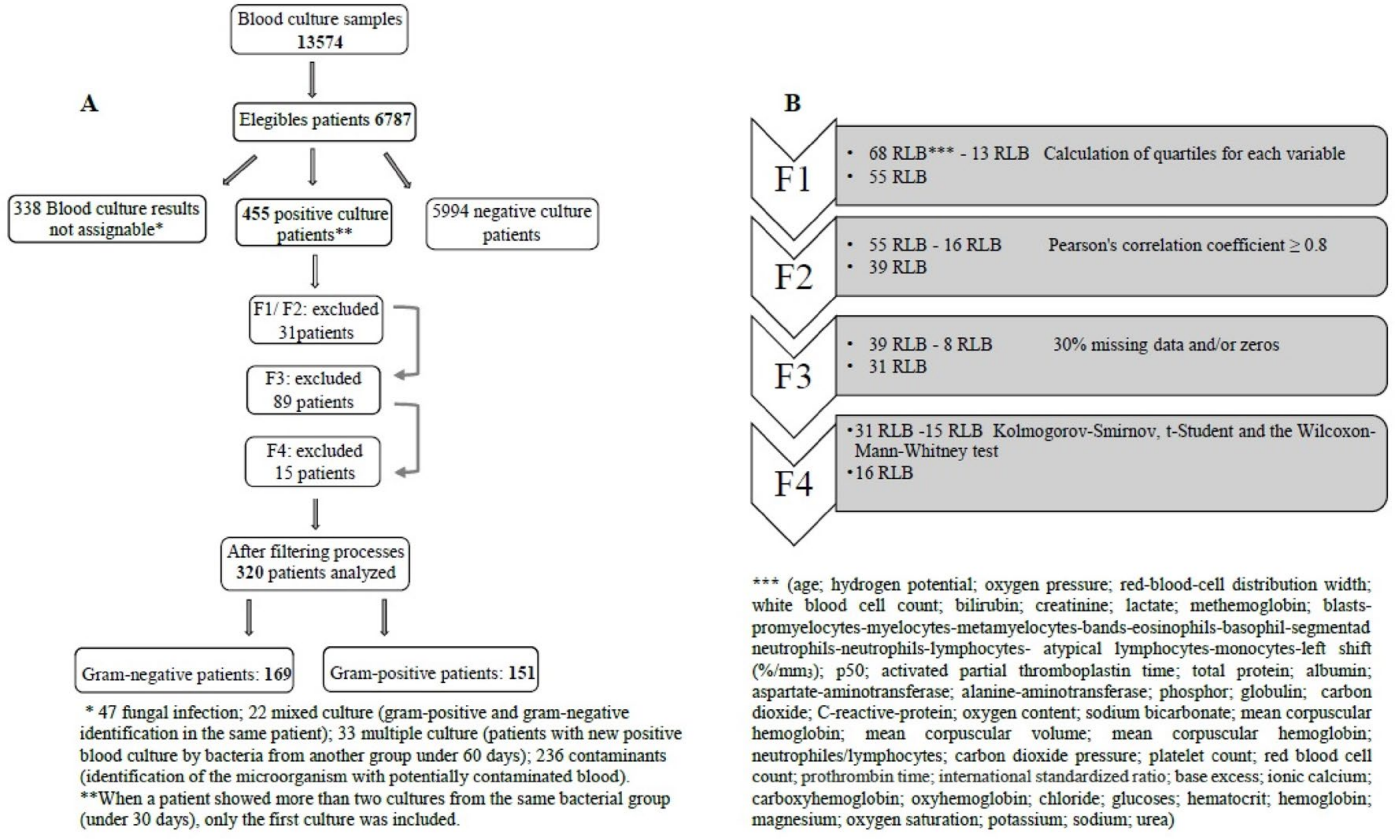
(**A**) Criterium for exclusion of patients; (**B**) exclusion criterium for routine laboratory biomarkers (RLB). F1 = first filtering process variables with different quartiles were kept in the bank at 0%, 25%, 50%, 75% and 100%. F2 = second filtering process, Pearson's correlation coefficient of 0.8 in absolute value was adopted as the cutoff. F3 = third filtering process, variables with 30% or more of lost values and/or zeros were removed. F4 = fourth filtering process, The Kolmogorov–Smirnov tests, the t-Student test and the Wilcoxon-Mann–Whitney test were used and variables with p ≤ 0.1 in at least one of the tests were maintained.

Out of a total of 793 BC-positive patients, 455 patients were analyzed and criteria for selection of these patients are described in Fig. 1. The epidemiological characteristics, place of hospitalization and initial site of infection were described in Table 1.

**Table 1:** General characteristics of all eligible patients.

Parameter	Gram-positive (n = 217)	Gram-negative (n = 238)	*p*-value	OR (95% CI)
Age (years), median (interquartile range)	39 (0.3–66)	52 (21–69)	0.003*	

	%(n)	%(n)		
Until 1 year	28.5 (62)	17.7 (42)	0.006*	0.53 (0.34–0.83)
2–13 years	6.5 (14)	5.0 (12)	0.51	
14–17 years	1.0 (2)	1.3 (3)	0.73	
> 18 years	64 (139)	76 (181)	0.005*	1.78 (1.18–2.68)
**Gender**			0.44	
Female	40.6 (88)	44.1 (105)		
Male	59.4 (129)	55.9 (133)		
**Admission**				
Surgery clinic	5.1 (11)	9.0 (21)	0.12	
Medical clinic	11.1 (24)	15.5 (37)	0.16	
Pediatrics	4.0 (09)	6.7 (16)	0.23	
Emergency	38.7 (84)	36.0 (86)	0.57	
General ICU	13.8 (30)	16.8 (40)	0.38	
Pediatric ICU	8.3 (18)	8.0 (19)	0.90	
Neonatal ICU	19.0 (41)	8.0 (19)	< 0.001*	0.37 (0.20–0.66)
**Initial infections site**				
Pulmonary	31 (67)	32.7 (78)	0.027*	1.09 (0.73–1.62)
Intra-abdominal	8.0 (20)	20.2 (48)	< 0.001*	2.48 (1.43–4.41)
Urinary tract	6.0 (12)	13.9 (33)	0.003*	2.74 (1.39–5.65)
Skin and soft tissue	22.0 (48)	8.4 (20)	< 0.001*	0.32 (0.18–0.56)
CNS	4.0 (08)	1.7 (04)	0.18	
Endocarditis	3.2 (07)	0.8 (02)	0.07	
Others or unknown	25.3 (55)	22.3 (53)	0.44	
**Mortality**				
Yes	30.0 (65)	37.4 (89)	0.09	
**Mortality initial infections site**				
Pulmonary	43.3 (29)	37.1 (29)	0.71	
Intra-abdominal	35.0 (07)	52.1 (25)	0.002*	3.5 (1.53–8.92)
Urinary tract	25.0 (04)	27.3 (09)	0.22	
Skin and soft tissue	27.1 (12)	30.0 (06)	0.10	
CNS	37.5 (01)	25.0 (01)	0.95	
Endocarditis	28.6 (02)	50.0 (01)	0.51	
Others or unknown	51.7 (09)	57.5 (18)	0.30	
Sepsis	36.0 (78)	29.0 (69)	0.71	
Septic shock	14.0 (30)	15.0 (35)	0.78	
No sepsis or septic shock	50.0 (109)	56.0 (134)	0.22	

Data given the percentage and numbers of patients (n), Two-Sample t-Student test or Chi square Test, OR = Odds ratio (95% Confidence Interval). ICU = Intensive care unit, CNS = Central Nervous System.

*Values of *p* < 0.05 were considered statistically significant.

Within the study population, the largest number of patients identified with bacteremia was from the emergency department (ED) totaling 170/455 (37.3%) (Table 1).

In terms of frequency, the most common initial infectious focus was pulmonary, totaling 145/455 (32%), followed by abdominal focus and skin/soft tissue, both with 68/455 (15%) each. The mortality rate was 154/455 (34%) in patients with BSI (Table 1).

The microorganism most frequently detected in BSI-GP was *Staphylococcus aureus* (95/217–43.7%), and the most prevalent BSI-GN was *Escherichia coli* (63/238–26.5%) as shown in Table 2. Regarding the mortality observed for the different bacteria, the following stand out: *Acinetobacter baumannii* with 55%, *Pseudomonas aeruginosa* 51.5%, Enterobacteria 34% and *S. aureus* with 30.4% of patients.

**Table 2:** Microorganisms isolated from patients with positive blood cultures Gram-negatives or Gram-positives.

Microorganism	n	Total isolates %	Bacterial group %
Gram-negative	238	52.3	
*Escherichia coli*	63	13.8	26.5
*Klebsiella* sp.*	52	11.4	21.8
*Pseudomonas* sp.**	37	8.1	15.5
*Acinetobacter baumannii*	20	4.4	8.4
*Enterobacter* sp.***	18	4.0	7.6
Others	48	10.6	20.2
Gram-positive	217	47.7	
*Staphylococcus aureus*	96	21	44.2
*Staphylococcus epidermidis*	42	9.2	19.4
*Enterococcus faecalis*	22	4.8	10.1
*Streptococcus pneumoniae*	15	3.3	6.9
*Staphylococcus* sp.	19	4.2	8.8
*Streptococcus* sp.	13	3.0	6.0
Others	10	2.2	4.6

*Includes 33 *P. aeruginosa 4 P. Putida.*

**Includes 47 *K. pneumoniae* 5 *K. Oxytoca.*

***Includes 17 *E. cloacae* 1 *E. aerogenes.*

### Single variable evaluation

**Figure 2:**
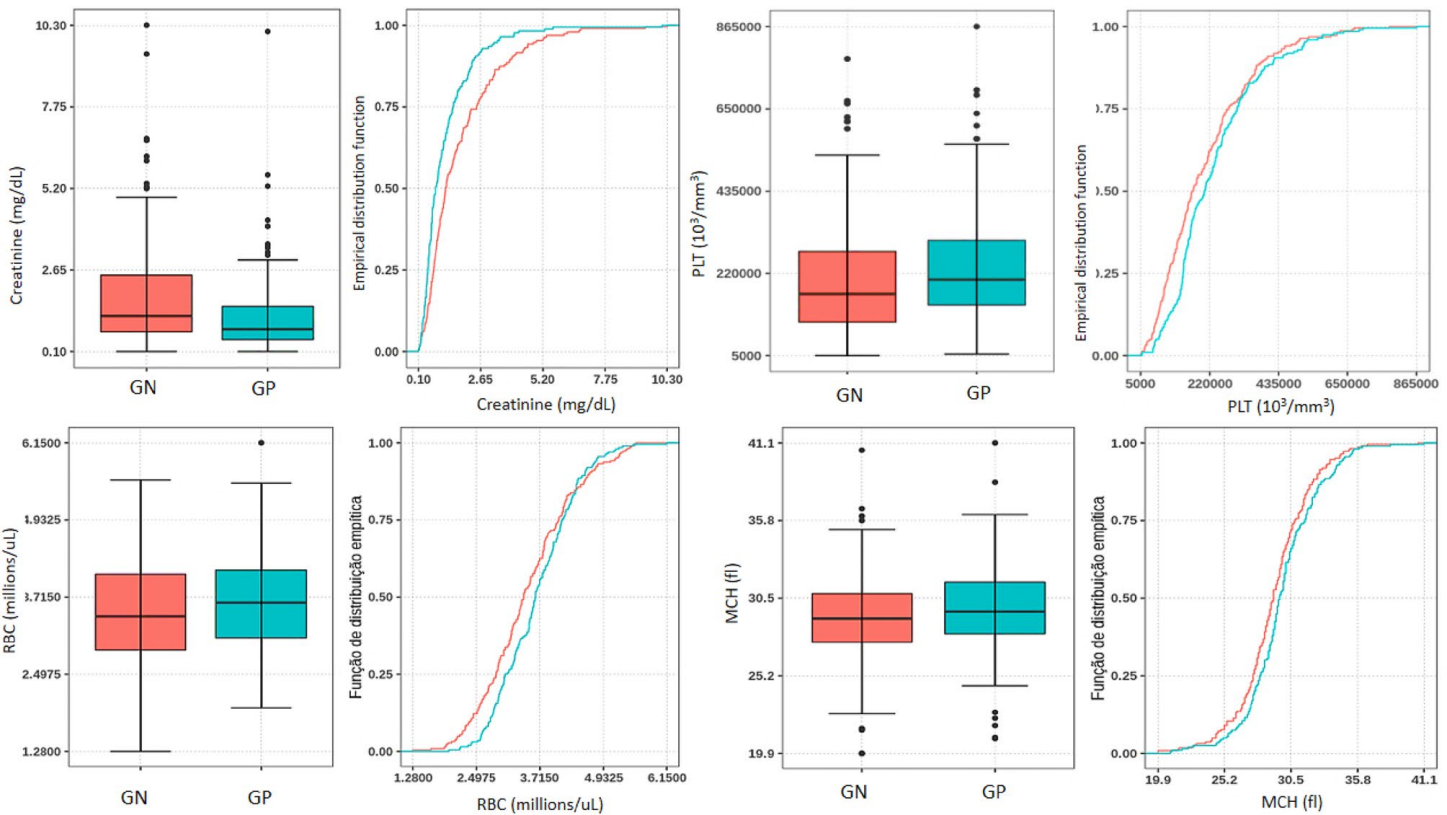
Comparisons of levels of single biomarkers stratified by patient categories. GN = Gram-negative (red); GP = Gram-positive (blue); PLT = platelet count; RBC = red blood cell count; MCH = mean corpuscular haemoglobin.

In the univariate analysis, RLB collected at the same time as the BC such as creatinine, platelet count (PLT), red blood cell count (RBC) and mean corpuscular hemoglobin (MCH) presented p ≤ 0.05 and AUC values of 0.63, 0.57, 0.56 and 0.56, respectively when comparing BSI-GN with BSI-GP, as shown in Fig. 2 and Supplementary Table S1.

### Multivariable prediction

After applying the filters indicated in Fig. 1 and Table 3, the remaining number of patients with BSI-GP was 151 and 169 with BSI-GN, totaling 320 patients and 16 RLB (Table 4), these variables formed the CM which was utilized in the prediction model to describe BSI-GP and BSI-GN. After excluding the RLBs that presented p > 0.05 in the multivariate analysis of the MC, the RM was obtained where the variables PLT, creatinine, MCH and RBC were kept (Table 4).

**Table 3:** Characteristics of all patients and laboratory biomarkers analyzed.

Biomarkers	Bacterial group	Selection of biomarkers	Statistical methods						
*n*	*Gram-positive (n)*	*Gram-negative (n)*	*F1 (nv)*	*F2 (nv)*	*F3 (nv)*	*F4 (nv)*	*KS*	*t-Student*	*WMW*
68	*217*	*238*	55	39	31	16	*p-value*		
Gender %(n)	F 40.6 (88) M 59.4 (129)	F 44.1 (105) M 55.9 (133)							
Age	39 (0.3–66)	52 (21–69)	X	X	X	X	0.01	0.003	0.002
ALAT (U/L)	42 (28–72)	31 (43–92)	X	–	–	–			
Albumin (G/dL)	2.15 (2–2.8)	2.15 (1.8–2.5)	X	X	–	–			
aPTT (sec)	34.9 (29.5–40.8)	38.8 (31–47.9)	X	X	–	–			
ASAT (U/L)	46 (26–91)	54.5 (28–119)	X	–	–	–			
Atypical lymphocytes (/mm^3^)	0 (0–0)	0 (0–0)	–	–	–	–			
Atypical lymphocytes (%)	0 (0–0)	0 (0–0)	–	–	–	–			
Base excess (mmol/L)	− 3.3 (− 7.1–0.3)	− 4.9 (− 10.5–0)	X	–	–	–			
Bands (/mm^3^)	708.5 (150–2017)	835 (262–2266)	X	X	X	X	0.37	0.07	0.16
Bands (%)	6 (2–13.5)	9 (2–19)	X	X	X	X	0.04	0.03	0.02
Basophil (/mm^3^)	0 (0–0)	0 (0–0)	–	–	–	–			
Basophil (%)	0 (0–0)	0 (0–0)	–	–	–	–			
Blasts (/mm^3^)	0 (0–0)	0 (0–0)	–	–	–	–			
Blasts (%)	0 (0–0)	0 (0–0)	–	–	–	–			
Ionized Calcium (mg/dL)	4.72 (4.44–5.07)	4.65 (4.34–4.91)	X	X	X	X	0.04	0.004	0.01
Carboxyhaemoglobin (%)	1.4 (1–1.7)	1.4 (1–1.8)	X	X	X	–	0.78	0.92	0.61
Chloride (mg/dL)	108 (104–112)	108 (104–113)	X	X	X	–	0.97	0.76	0.86
CO_2_ total (mmol/L)	21.8 (18.3–26-1)	20.8 (16–25.3)	X	X	X	X	0.04	0.02	0.04
Creatinine (mg/dl)	0.8 (0.48–1.5)	1.21 (0.71–2.49)	X	X	X	X	< 0.001	< 0.001	< 0.001
CRP (mg/dl)	16.5 (5.9–25.8)	18.1 (5.6–27.4)	X	X	X	–	0.71	0.51	0.62
ctO_2_ (mL/dL)	14.1 (12.1–16.7)	12.9 (10.7–15.5)	X	–	–	–			
Phosphor (mg/dl)	4.6 (4–5.25)	4.1 (2.9–6.1)	X	–	–	–			
Eosinophils (/mm^3^)	99.3 (0–263)	46.2 (0–167.5)	–						
Glucoses (mg/dl)	122 (98–182.5)	120 (92–165.5)	X	X	X	–	0.65	0.17	0.43
Globulin	2.55 (2.1–3)	2.6 (2.2–3.25)	X	–	–	–			
Haematocrit (%)	32.2 (27.6–36.7)	29.9 (24.9–34.7)	X	–	–	–			
Haemoglobin (G/dL)	10.8 (9.1–12.4)	9.8 (8.2–11.9)	X	–	–	–			
Reduced Haemoglobin	3 (1.5–6.1)	3.7 (1.9–6.2)	X	X	X	–	0.69	0.39	0.32
HCO_3_ (mmol/L)	21.9 (18.4–24.8)	20.75 (16–24.4)	X	–	–	–			
Lactate (mmol/L)	1.8 (1.2–2.8)	2.4 (1.5–4.4)	X	X	X	X	0.01	0.001	< 0.001
Left shift (/mm^3^)	822 (152–2129.5)	917 (285–2555)	X	–	–	–			
Left shift (%)	7 (2–15.5)	10 (3–23)	X	–	–	–			
Lymphocytes (/mm^3^)	1205 (686.4–2132)	1043 (6750–19,340)	X	X	X	–	0.4	0.12	0.15
Lymphocytes (%)	10 (5–21)	10 (5–19)	X	X	X	–	0.96	0.71	0.60
Magnesium (mg/dl)	1.9 (1.6–2.1)	1.8 (1.5–2.2)	X	X	–	–			
MCH (fl)	29.6 (28–31.6)	29.1 (27.5–30.8)	X	X	X	X	0.06	0.02	0.02
MCHC (g/dl)	33.4 (29.9–36.2)	33.2 (28.8–36.2)	X	X	X	X	0.02	0.16	0.07
MCV (pg)	88.4 (84.8–93.5)	87.7 (83.4–92.8)	X	–	–	–			
Myelocytes (/mm^3^)	0 (0–0)	0 (0–0)	–	–	–	–			
Myelocytes (%)	0 (0–0)	0 (0–0)	–	–	–	–			
Metamyelocytes (/mm^3^)	0 (0–75.5)	0 (0–126)	–	–	–	–			
Metamyelocytes (%)	0 (0–1)	0 (0–1)	–	–	–	–			
Methaemoglobin (%)	0.9 (0.7–1.3)	1.1 (0.8–1.4)	X	X	X	X	0.27	0.03	0.09
Monocytes (/mm^3^)	696 (331–1195)	511 (230–1044)	X	X	X	X	0.007	0.005	< 0.001
Monocytes (%)	6 (3–8)	4 (2–7)	X	X	X	X	0.03	0.04	0.01
NLCR	7.7 (3.4–17.8)	8.5 (3.8–18.4)	X	X	X	–	0.76	0.91	0.42
Neutrophiles (/mm^3^)	10,295 (5562–15,610)	9405 (4924–65,768)	X	–	–	–			
Neutrophiles (%)	81 (70–89)	84 (73–91)	X	X	–	–			
Oxihaemoglobin (%)	94.7 91.1–96.2)	94 (91–95.5)	X	–	–	–			
Oxygen Saturation (%)	96.9 (93.7–98.4)	96.2 (93.6–98)	X	–	–	–			
pCO_2_ (mmHg)	35.5 (30.2–42.4)	34.6 (29.3–42.6)	X	X	X	–	0.96	0.95	0.63
pH	7.39 (7.31–7.45)	7.38 (7.25–7.44))	X	X	X	X	0.14	0.006	0.06
PLT (10^3^/mm^3^)	204 (137–306)	166 (92–279)	X	X	X	X	0.001	0.03	0.008
pO_2_ (mmHg)	88.3 (68.8–121.1)	86.4 (71–118.5)	X	X	X	–	0.84	0.14	0.73
Potassium (mmol/L)	3.9 (3.4–4.4)	3.8 (3.4–4.5)	X	X	X	–	0.79	0.45	0.81
Promyelocytes (/mm^3^)	4.54 (0–756)	18.7 (0–1970)	–	–	–	–			
Promyelocytes (%)	0.015 (0–2)	0.03 (0–2)	–	–	–	–			
*p*50 (mmHg)	25.5(23.8–27.6)	26.5 (24.5–31)	X	X	X	X	0.004	< 0.001	0.003
RBC (millions/uL)	3.6 (3–4.2)	3.41 (2.9–4.1)	X	X	X	X	0.02	0.02	0.02
RDW (%)	15.4 (14–117)	15.6 (14.3–17.4)	X	X	X	–	0.60	0.14	0.29
Segmented neutrophil (/mm^3^)	8644 (4748–13,377)	7837(3698–14,544)	X	X	X	–	0.47	0.61	0.37
Segmented neutrophil %	70 (56–79)	69 (53–80)	X	X	X	–	0.32	0.60	0.64
Sodium (mmol/L)	136 (133–140)	137 (133–140)	X	X	X	–	0.90	0.81	0.75
TAP (%)	69.7 (51.7–85)	58.1 (38.8–69.9)	X	X	–	–			
TAP-INR	1.2 (1.1–1.5)	1.4 (1.24–1.8)	X	X	–	–			
TP (G/L)	4.9 (4.1–5.2)	4.8 (4.2–5.6)	X	X	–	–			
Urea (mg/dL)	53.5 (33–91)	67.5 (40–119)	X	X	–	–			
WBC (/mm^3^)	12.5 (7.9–18.4)	12.4 (6.8–19.3)	X	–	–	–			

X: selected biomakers; –: non selected biomakers; n = number of samples; nv = number of variables. Data given as mean with interquartile range (Q1, Q3).

*KS* two-sample Kolmogorov–Smirnov test, *t-Student* Two-Sample t-Student test, *WMW* Two Sample Wilcoxon Signed Rank Test, *ALAT* alanine aminotransferase, *aPTT* activated partial thromboplastin time, *ASAT* aspartate aminotransferase, Left shift (bands + metamyelocyte + myelocyte + promyelocyte + blasts), *CO*_*2*_ carbon dioxide, *CRP* C-reactive protein, *ctO*_*2*_ oxygen contend, *HCO*_*3*_ sodium bicarbonate, *MCH* mean corpuscular haemoglobin, *MCV* mean corpuscular volume, *MCHC* mean corpuscular hemoglobin, *NLCR* Neutrophils/Lymphocytes count ratio, *pCO*_*2*_ carbon dioxide pressure, *pH* hydrogen potential, *PLT* platelet count, *pO*_*2*_ oxygen pressure, *RBC* red blood cell count, *RDW* red blood cell distribution width, *TAP* prothrombin time, *INR* International standardized ratio, *TP* total protein, *WBC* white blood cell count.

Variable filtering process: F1 = variables with different quartiles were kept in the bank at 0%, 25%, 50%, 75% and 100%. F2 = Pearson's correlation coefficient and 0.8 in absolute value was adopted as the cutoff, variables correlated with each other were excluded. F3 = variables with a maximum of 30% of lost values or values equal to zero. F4 = variables with *p value* ≤ 0.1 in at least one of the tests were maintained.

**Table 4:**
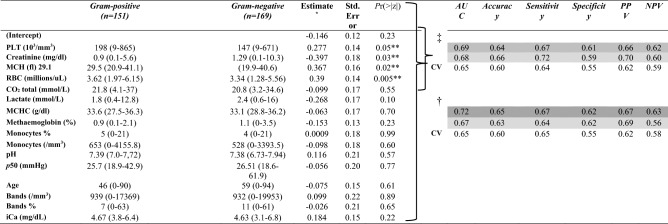
Estimated coefficients for each selected variable. Adjustment, Validation and Prediction, considering the previously filtered variables (16) for performance in the prediction of Gram-negative bacteremia.

Data given as mean (minimum and maximum values).

*n* number of samples, *iCa* ionized calcium, *CO*_*2*_ carbon dioxide, *MCH* mean corpuscular haemoglobin, *MCHC* mean corpuscular haemoglobin, *pH* hydrogen potential, *PLT* platelet count, *RBC* red blood cell count.

^†^Model Complete.

Estimate*: logistic regression coefficients estimates.

**Only the variables are statistically significant at a 5% significance level (^‡^Model Reduced). AUC = area under curve PPV = Positive Predictive Value NPV = Negative Predictive Value CV = coefficient of variation. Accuracy, Sensitivity, Specificity, PPV and NPV estimates were obtained considering a cutoff value equal to 0.50. Dark grey: Validation *(n* = *320)*. Light grey: Prediction using the 2019 database *(n* = *69)*. The coefficients estimated (Std. Error) for Creatinine, MCH, PLT and RBC in the Reduced Model are—0.53 (0.14), 0.34 (0.13), 0.35 (0.13) and 0.36 (0.13) respectively.

**Figure 3:**
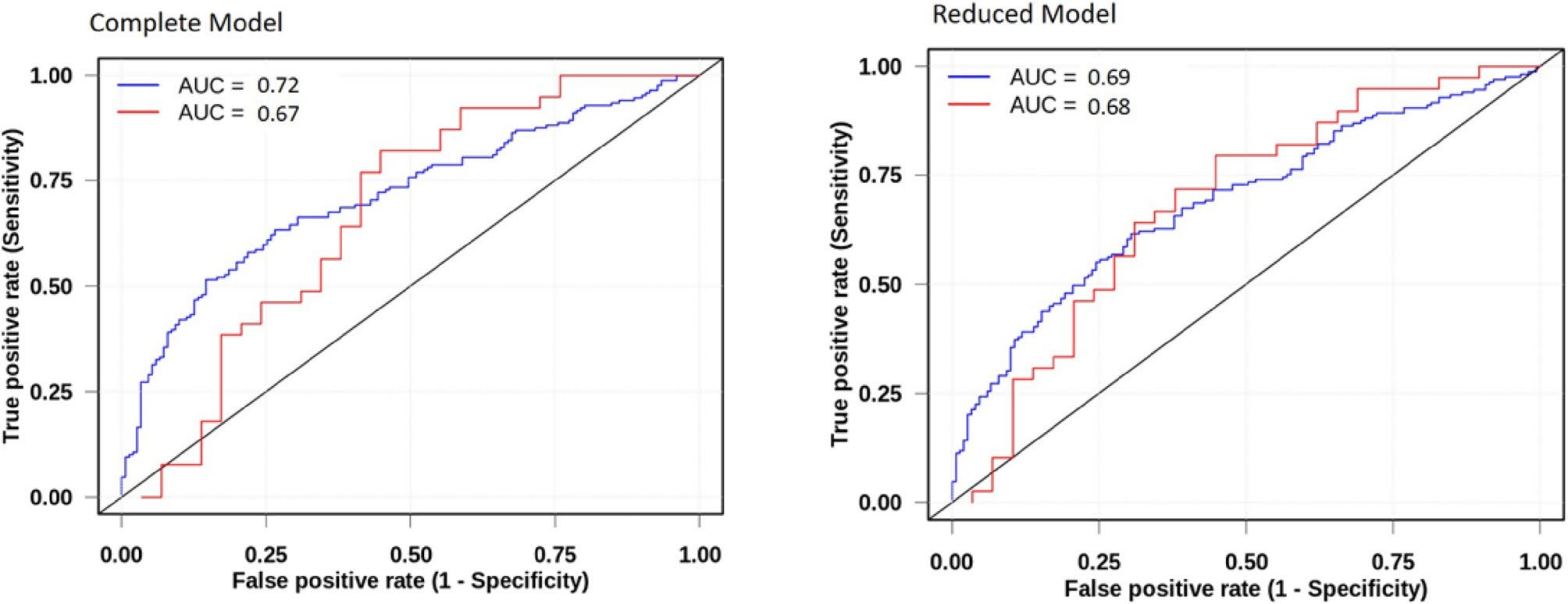
Comparison between the Complete and Reduced Models to predict Gram-negative bacteremia. AUC = area under curve. Complete Model = Monocytes%, Bands (/mm^3^), p50 (mmHg); Mean Corpuscular Haemoglobin (CHCM) (g/dl), Bands%, Age, Monocytes (/mm^3^), Hydrogen potential (pH), total CO_2_ (mmol/L), Methaemoglobin (%), ionized Calcium (iCa—mg/dL), Lactate (mmol/L), platelet count (PLT—10^3^/mm^3^), Creatinine (mg/dl), Mean corpuscular haemoglobin (MCH—fl) and Red blood cell count (RBC—millions/μL). Reduced Model = PLT, creatinine, MCH and RBC. Blue: Validation *(n* = *320)*. Red: Prediction using the 2019 database *(n* = *69)*.

To predict bacteremia by GN bacteria, from the mean value of the tenfold-cross-validation repeated 10-times and using the optimistic model, we obtained estimates of AUC of 0.72, accuracy of 0.65, sensitivity of 0.67 and specificity of 0.62 using CM and AUC of 0.69, accuracy of 0.64, sensitivity of 0.67 and specificity of 0.61 using the RM (Table 4 and Fig. 3).

Other methods, such as the backward-selection and stepwise-selection, presented the same variables as the RM to predict BSI-GN. The covariates selected by RFE were: iCa, creatinine, MCH, RBC, lactate, methemoglobin and PLT, establishing a model with a 0.71 AUC. Using Boruta, the variables selected were iCa, creatinine, RBC, age and PLT, resulting in a model with a 0.67 AUC.

The models (CM and RM) were checked against a new-database that was not used to adjust the models. This database included BC data from 2632 samples (1316 patients), of which 1191 patients presented negative BC. Among the 125 patients with positive BC, 57 were excluded (exclusion criteria already added to the validation model), remaining 68 patients, 43% (29/68) with BSI-GP and 57% (39/68) with BSI-GN. In this test of the CM predictive model, an AUC of 0.67 was obtained, resulting in a sensitivity of 0.62, specificity of 0.64. The RM prediction presented the following estimates: AUC of 0.68, sensitivity of 0.59 and specificity of 0.72 for detection of BSI-GN. By the likelihood ratio test, the RM was not statistically different from the CM (p = 0.64) (Table 4 and Fig. 3).

## Discussion

The knowledge of the bacterial group involved in the bacteremia process favors the early initiation of the most appropriate empirical therapy and increases patients' chances of survival^4^. Although many RLB have been reported as predictors of bacteremia, sepsis or mortality, they are not commonly used to distinguish between BSI-GN or BSI-GP^5,6,8,10,11,15–17^. Of the 68 RLB evaluated in this study, we set up a new statistical model with 4 covariates, predicting BSI-GN with an AUC of 0.69 a precision of 0.64, a sensitivity of 0.61 and a specificity of 0.67, which could assist clinicians in choosing the antimicrobial before the final BC result (Table 4, Fig. 3).

This model involves multiple analyzes where the predictor variables are analyzed simultaneously, so that the effect of each variable is adjusted to the effect of the others. Some of these biomarkers have already been proposed in other studies to predict BSI or the bacterial group involved in BSI, but few have shown good sensitivity and specificity as an independent test^9,11,14,17^.

Regarding some patient’s data included in the study, the inclusion of the pediatric population is a controversial subject. While Bash et al*.* demonstrated that the large differences in the immunological system would make this population special, Oksuz et al. and Colak et al., when using pediatric patients in the proportion of 12.5 and 35%, respectively, indicated that the inclusion of children and adults was beneficial in their studies^1,7,18^. We maintained the pediatric population (28.6% in this study) and agreed with Colak et al., that age inclusion may have contributed to reducing the bias of the selected group, since age in our study was considered a covariate, which was related to other biomarkers in all statistical analyses (Table 3).

In this study, the prevalence of BSI-GP was 47.7% and BSI-GN 52.3% (Tables 1, 2). Among the BSI-GN, species of the Enterobacteriaceae family were the most prevalently represented, mainly by *E. coli* (26.5%) and *Klebsiella pneumoniae* (19.7%). We highlight the high mortality associated with non-glucose fermenting GN bacilli species such as *P. aeruginosa* 51.5% and *A. baumannii* 55%. Among BSI-GP, the highest frequency was *S. aureus* (43.7%), whose mortality was 30.4%. The present data corroborates the literature^9,11,19–21^, demonstrating a greater difficulty in the treatment of BSI-GN, sometimes related to the specific characteristics of this bacterial group, such as the known lipopolysaccharide with an endotoxin specific to GN^21–23^; or the higher resistance to antibacterial drugs of clinical use, mainly found in *A. baumannii* and *P. aeruginosa,* which increases their mortality rate^24,25^.

Thus, if the RLB data could predict the bacterial group involved in BSI, even with an estimated 70% accuracy, it would be extremely useful in initiating a more targeted empirical therapy while blood culture results are not yet available to the physician, which can take an average of 2–3 days^5–7^.

Levy, 2017 demonstrated that prior knowledge of the presence of an infectious focus can help to report the infectious agent in BSI^26^. In this study, the initial infectious focus of the abdomen presented OR 2.48 (1.43–4.41) p < 0.001, predicting BSI-GN as well as, the urinary focus that presented moderate risk for BSI-GN with OR 2.74 (1.39–5.65) p = 0.003 (Table 1).

In our study, mortality between BSI-GP and BSI-GN only showed a statistically significant difference when the initial focus was intra-abdominal (p = 0.002). The clinical parameters added to the initial infection focus, in association with RLB parameters, may increase the chance of predicting the bacterial group in BSI, helping to choose the most appropriate antimicrobial treatment and thus contributing to a reduction in morbidity and mortality.

Unexpectedly, the ED had the highest number of BSI (37.3%), followed by the ICU, with only 15.4%. Wang et al. also found a high frequency of BSI in ED due to delays in the identification of BSI etiologic agents and the initiation of the correct antimicrobial therapy, which impair the patients' prognosis^12,27–31^.

When individually analyzed, some RLB showed statistical differences in univariate analysis, such as creatinine (p < 0.001), PLT (p < 0.03), RBC (p < 0.02), MCH (p < 0.02). However, the values of AUC for creatinine, PLT, RBC and HCM (0.63, 0.57, 0.56, 0.56, respectively), suggest that biomarkers, separately, are not able to differentiate the bacterial group in BSI satisfactorily. Indeed, their values have a large standard deviation, demonstrating that these RLB do not react in the same way for all patients (Table S1, Fig. 2). Ratzinger et al. obtained a predictive model composed of seven parameters (AUC of 0.67), which was significantly better (p ≤ 0.001) than the best individual predictive parameter (AUC of 0.589^11^. Ljunsgstrom et al. determined a predictor model for bacteremia composed of four biomarkers with high AUC (0.78), and that also was significantly higher (all p < 0.001) than the composite three-biomarker (AUC of 0.75) and all single biomarkers separately, except procalcitonin (PCT) (p = 0.06)^8^.

Here we present, for the selection of variables, a new selection model capable of discriminating bacterial groups in BSI by using four sequential filters (Fig. 1). Then, we present a predictor model with 16 RLBs (CM) that showed an AUC of 0.72, which is slightly higher than the AUC of the RM (0.69) composed of only four RLB easily performed in most laboratories. The CM and RM had sensitivity of 0.62 and 0.61, respectively, and specificity of 0.67 for both (Table 4 and Fig. 3). The CM and RM are two hypotheses with equal efficiencies and, according to the principle of parsimony, which advocates the simplest, RM would be more easily used, allowing faster interpretation and lower cost.

The LR models constructed with automatic variables already described in the literature, such as forward selection, backward selection, stepwise selection, RFE and Boruta feature selection, do not outperform the values of the metrics estimated by the RM here proposed^32^.

Tang et al*.* determined specific combinations involving lymphocyte count, PLT, neutrophil-to-lymphocyte ratio (NLCR), mean platelet volume (MPV), MPV-to-PLT ratio (MPV/PLT), platelet-to-larger cells ratio (P-LCR), and C-reactive-protein (CRP), and obtained a good ability to distinguish various pathogens in BSI from negative BC. The highest AUC of their study was for BSI-GP, of 0.715, and 0.777 for BSI-GN, with 0.797 for *E. coli* BSI, 0.943 for *Enterobacter aerogenes* BSI, 0.830 for *P. aeruginosa* BSI and 0.767 for *S. aureus* BSI^14^. Our work was carried out among patients with confirmed BSI by GN or GP, and did not include patients with negative BC, so that changes in RLB were greater in both groups, which reduced the difference between them and may explain the lower values of AUC here obtained. When Tang et al*.* compared BSI-GP with BSI-GN, the highest AUC obtained was 0.63, while our CM and RM presented AUC of 0.71 and 0.69 respectively. Our work evaluated the RLB at group level, not at species. Although PLT was one of the biomarkers selected in our statistical model, the MPV and P-LCR parameters that evaluate the platelet series are not part of the RLB routine at the studied hospital.

Biomarkers such as PCT, lipopolysaccharide-binding protein, the CD14-ST isoform and the interleukin-6 measurement are described as potential biomarkers to distinguish BSI-GN, however, these data are inconsistent, since some studies obtained promising results with PCT^1,12,33,34^. This is contrary to Ruddel et al., who found a low discriminatory power of the PCT to guide therapeutic decisions^9,35,36^. Our study was based on RLB, and therefore did not evaluate the aforementioned tests. This fact could be considered a limitation since they presented promising results; however, these markers are not part of the routine of most clinical laboratories.

The retrospective nature of the study may introduce bias in the analysis of the results. We worked with a set of data to generate the predictive model, and with another set of data to test it, so although we believe that our model is validated, it must be applied in other health institutions and its applicability still needs to be tested in clinical practice. We emphasize that the models should not be applied to results obtained after blood transfusions and electrolyte replacement.

Challenging the proposed model with unused data to develop the predictive model has not been a common practice yet in literature. The model built in our study was tested with a database that was not part of the model's validation. The forecast values obtained for the CM (AUC of 0.67) and for the RM (AUC of 0.68) confirmed the discriminatory capacity of the model developed for the BSI-GN and demonstrated that the two models are similar (Table 4 and Fig. 3).

It would be ideal to propose a model with high specificity for the indication of BSI-GN or BSI-GP, so that we could indicate high and low values for creatinine, PLT count, RBC and MCH. However, as we chose to do a robust study with multiple variables, our specificity was around 70%. As described in Table 3 and Fig. 1, we can see that the values referring to creatinine, PLT count, RBC and MCH for both BSI-GN and BSI-GP have important differences in the medians. For example, creatinine values for BSI-GN are generally higher for BSI-GN than for BSI-GP (median of 1.66 for BSI-GN and 0.8 for BSI-GP) as well as for PLT count, conversely, for BSG-GN the values are generally lower than for BSI-GP (median for BSI-GN of 166 and for BSI-GP of 204). However, as described in the text and seen in Fig. 1 and Table 3, the standard deviation is high and therefore we cannot establish a cut-off point that indicates BSI-GP or BSI-GN. These deviations are inherent to the unique characteristics of each patient, demonstrating that univariate analyzes are inadequate, since these differences can only be minimized or corrected with robust statistical models.

In conclusion, our proposed model using four RLB, easy to obtain, could be used daily without additional costs (creatinine, PLT, RBC and MCH) and can be an early warning system in at least 13 h to detect the bacterial group of the etiological agent causing BSI, through a simple computer system or even a cellphone app. We believe that the association of this RM along with the patient's clinical data could increase the chance of success in finding the bacterial group involved in the BSI, and thus assist in the management of antimicrobial therapy in a more accurate way. It is also important to add that the use of these models can help in decision making for empirical therapy without ever forgetting that the empirical prescription of antimicrobials must also consider the specificities of each patient.

## Methods

### Study design and data collection

This retrospective study included patients that had BSI between 2013 and 2018. The study was conducted at the Maringá University Hospital, in Maringá, Brazil, which is a public teaching general hospital. This hospital does not perform transplants or treatments for cancer patients. The data from BC tests (collected in duplicates) and RLB were analyzed, both collected on the same day. BC and bacterial identification was performed in the BACTEC™ and Phoenix™ systems (BD Diagnostic Systems, Sparks, MD), respectively. Complete blood cell counts were determined using the Sysmex-XE-2100™ (Sysmex-Corporation, Kobe, Japan). Biochemical tests were performed using VITROS™ 5.1-FS (Ortho-Clinical Diagnostics, New Jersey, USA). Gasometric, electrolytes, ionized calcium (iCa), glucose, lactate and creatinine were measured with ABL800 ™ FLEX (Radiometer, Copenhagen, Denmark). Coagulation tests were analyzed using the ACL™ Elite-Pro (Beckman-Coulter, California, USA).

The inclusion criteria were: patients with positive BC with GN or GP bacteremia present in both collected samples, or when at least one BC was positive with pathogens of clinical interest. Exclusion criteria were as suggested by Hall et al. (Fig. 1)^37^. All study data were obtained from the hospital database.

### Statistical analysis

The evaluated data were organized in the Microsoft-Excel^®^ 2007 software. The patient’s general characteristics were analyzed using the Odds ratio (OR), Student’s t-test or chi-square-test, and values of p ≤ 0.05 were considered statistically significant.

For the construction of a predictive model, the variables were selected sequentially by applying four filters (“R codes Supplementary data”), as shown in Fig. 1 and Table 2. Thus, 320 patients and 16 variables (Complete Model-CM) remained to be tested in the prediction model. Using only the statistically significant covariates (p ≤ 0.05) of the CM, we obtained a Reduced-Model (RM) composed of four variables.

The logistic-regression (LR) model was constructed taking as a response variable the classification of GP or GN and a series of biomarkers as covariates. In the model fit, the presence of GN was defined as an event. Its predictive capacity was assessed by the area under the curve ROC-AUC, considering ten-fold-cross-validation repeated ten-times, as well as the values for sensitivity, specificity, positive predictive value (PPV), and negative predictive value (NPV) along with their 95% CIs. In addition, other selection methods (direct-selection, reverse-selection, stepwise-selection, recursive elimination of features-RFE and Boruta) were also applied^32^.

The proposed models (CM and RM) were checked for their applicability using a different database (BC and RLB 2019).

### Ethical considerations

The present study was approved by the ethics committee for research with human beings “Comitê Permanente de Ética em Pesquisa com Seres Humanos” (COPEP) of the State University of Maringá, number 2093342, in accordance with the national ethics committee Resolution 466/12—MS (Ministry of Health). The approval was also carried out by the internal research ethics committee of the University Hospital of Maringá by the. “Comissão de Regulamentação de Atividades Acadêmicas” (COREA), number 0447/2017. It was conducted in accordance with the Declaration of Helsinki. All confidentiality was guaranteed in the processing of data and, for this, the participants were assigned a consecutive identification number that guaranteed anonymity. The informed consent was waived, according by the local and national research ethics committees above mentioned.

## Supplementary Information

Supplementary Information 1.

Supplementary Information 2.

Supplementary Table S1.

## Data Availability

A table as supplementary material containing the raw data that were used in the study was attached. Further information about the data and conditions for access to anonymized data can be requested from the corresponding author.
