# Supplementary material for: Routine laboratory biomarkers used to predict Gram-positive or Gram-negative bacteria involved in bloodstream infections
Source: Sci Rep. 2022 Sep 14;12:15466. doi: 10.1038/s41598-022-19643-1 (PMC9474441; doi:10.1038/s41598-022-19643-1)
Supplement: Supplementary file 1 — Supplementary Information 1. [file 41598_2022_19643_MOESM1_ESM.pdf]

R codes

Code ▾

Hide

```
knitr::opts_chunk$set(fig.path="/home/jmazucheli/Dropbox/Daniela2020/figuras_notebook_1/")
```

Hide

```
source("/home/jmazucheli/Dropbox/Daniela2020/preambulo.R")
```

Descriptive Statistics — Primary data set: 455 observations 70 variables

Hide

```
kable(todas, digits = 3)
```

variavel	n.validos	pct_validos	n.perdidos	pct_perdidos	pct_zeros	pct_zero_ou_perdidos	freqRatio	pe
linfocitos_a	422	92.747	33	7.253	0.000	7.253	1.000	
nlcr	422	92.747	33	7.253	0.000	7.253	1.000	
vcm	422	92.747	33	7.253	0.000	7.253	1.000	
vg	422	92.747	33	7.253	0.000	7.253	1.000	
hcm	422	92.747	33	7.253	0.000	7.253	1.200	
hemoglobina_p	422	92.747	33	7.253	0.000	7.253	1.182	
segmentados_r	422	92.747	33	7.253	0.000	7.253	1.250	
chcm	422	92.747	33	7.253	0.000	7.253	1.062	
neutrofilos_r	422	92.747	33	7.253	0.000	7.253	1.263	
linfocitos_r	422	92.747	33	7.253	0.000	7.253	1.464	
segmentados_a	421	92.527	34	7.473	0.000	7.473	1.000	
leucocitos	421	92.527	34	7.473	0.000	7.473	2.000	
plaquetas	421	92.527	34	7.473	0.000	7.473	1.200	
hemacias	421	92.527	34	7.473	0.000	7.473	1.000	
rdw	421	92.527	34	7.473	0.000	7.473	1.091	
neutrofilos_a	419	92.088	36	7.912	0.000	7.912	2.000	
monocitos_r	422	92.747	33	7.253	0.879	8.132	1.125	
monocitos_a	421	92.527	34	7.473	0.879	8.352	2.000	
idade	455	100.000	0	0.000	11.648	11.648	4.818	
calcio_ionizado	382	83.956	73	16.044	0.000	16.044	1.000	
potassio	382	83.956	73	16.044	0.000	16.044	1.045	
cloreto	382	83.956	73	16.044	0.000	16.044	1.160	
sodio	382	83.956	73	16.044	0.000	16.044	1.167	
creatinina	369	81.099	86	18.901	0.000	18.901	1.167	
glicose	355	78.022	100	21.978	0.000	21.978	1.000	
dav_r	422	92.747	33	7.253	14.725	21.978	2.310	
bastonetes_a	422	92.747	33	7.253	14.945	22.198	34.000	
dav_a	421	92.527	34	7.473	14.725	22.198	22.333	
bastonetes_r	422	92.747	33	7.253	14.945	22.198	2.125	
pcr	350	76.923	105	23.077	0.000	23.077	1.600	
po2	337	74.066	118	25.934	0.000	25.934	1.000	
pco2	337	74.066	118	25.934	0.000	25.934	1.000	

variavel	n.validos	pct_validos	n.perdidos	pct_perdidos	pct_zeros	pct_zero_ou_perdidos	freqRatio	pe
co2_total	337	74.066	118	25.934	0.000	25.934	1.200	
oxihemoglobina	337	74.066	118	25.934	0.000	25.934	1.100	
saturacao	337	74.066	118	25.934	0.000	25.934	1.200	
ph	337	74.066	118	25.934	0.000	25.934	1.056	
p50	336	73.846	119	26.154	0.000	26.154	1.333	
hco3	337	74.066	118	25.934	0.220	26.154	1.000	
cto2	336	73.846	119	26.154	0.000	26.154	1.000	
hemoglobina_reduzida	337	74.066	118	25.934	0.220	26.154	1.000	
lactato	336	73.846	119	26.154	0.000	26.154	1.333	
excesso_de_base	337	74.066	118	25.934	0.440	26.374	1.167	
carboxihemoglobina	337	74.066	118	25.934	0.440	26.374	1.071	
metahemoglobina	335	73.626	120	26.374	0.220	26.593	1.000	
eosinofilos_a	422	92.747	33	7.253	36.264	43.516	82.500	
eosinofilos_r	422	92.747	33	7.253	36.264	43.516	1.058	
ureia	222	48.791	233	51.209	0.000	51.209	1.600	
magnesio	186	40.879	269	59.121	0.000	59.121	1.048	
tap	147	32.308	308	67.692	0.000	67.692	1.200	
tap_rni	147	32.308	308	67.692	0.000	67.692	1.000	
ast_tgo	140	30.769	315	69.231	0.000	69.231	1.000	
alt_tgp	140	30.769	315	69.231	0.000	69.231	1.200	
kptt_seg	134	29.451	321	70.549	0.000	70.549	1.000	
meta_a	422	92.747	33	7.253	63.956	71.209	97.000	
meta_r	422	92.747	33	7.253	63.956	71.209	4.619	
mielocitos_a	422	92.747	33	7.253	79.121	86.374	180.000	
mielocitos_r	422	92.747	33	7.253	79.121	86.374	10.286	
basofilos_a	422	92.747	33	7.253	83.956	91.209	382.000	
prot_totais	40	8.791	415	91.209	0.000	91.209	1.333	
albumina	40	8.791	415	91.209	0.000	91.209	1.250	
basofilos_r	422	92.747	33	7.253	83.956	91.209	10.053	
globulina	38	8.352	417	91.648	0.000	91.648	1.000	
fosforo	19	4.176	436	95.824	0.000	95.824	1.500	
promielocitos_a	422	92.747	33	7.253	91.648	98.901	417.000	
promielocitos_r	422	92.747	33	7.253	91.648	98.901	104.250	
linfocitos_atipicos_a	422	92.747	33	7.253	91.868	99.121	418.000	
linfocitos_atipicos_r	421	92.527	34	7.473	91.648	99.121	417.000	
blastos_a	422	92.747	33	7.253	92.308	99.560	420.000	
blastos_r	422	92.747	33	7.253	92.308	99.560	420.000	

Percentiles — Primary data set

Hide

```
X     <-  entrada[, -c(1,2)]
gram  <-  entrada$gram
sexo    <-  entrada$sexo

qtl   <-  plyr::ldply(lapply(1:ncol(X), function(i) quantile(X[,i], na.rm = T)))
qtl$L <-    apply(qtl, 1, function(x) ifelse(length(unique(x)) == 5, "TRUE", "FALSE"))
rownames(qtl)   <-  names(X)
kable(qtl)
```

	0%	25%	50%	75%	100%	L
albumina	1.100000	1.8750	2.150000	2.500	3.90000	TRUE
alt_tgp	12.000000	29.0000	43.000000	82.000	1684.00000	TRUE
ast_tgo	11.000000	28.0000	51.500000	98.000	8013.00000	TRUE
basofilos_a	0.000000	0.0000	0.000000	0.000	621.10000	FALSE
basofilos_r	0.000000	0.0000	0.000000	0.000	4.00000	FALSE
bastonetes_a	0.000000	203.5000	805.000000	2127.000	19953.00000	TRUE
bastonetes_r	0.000000	2.0000	7.500000	17.000	63.00000	TRUE
blastos_a	0.000000	0.0000	0.000000	0.000	88641.00000	FALSE
blastos_r	0.000000	0.0000	0.000000	0.000	90.00000	FALSE
calcio_ionizado	3.080000	4.4100	4.675000	4.980	6.77000	TRUE
carboxihemoglobina	0.000000	1.0000	1.400000	1.700	4.50000	TRUE
chcm	27.500000	32.4000	33.400000	34.400	36.30000	TRUE
cloreto	86.000000	104.0000	108.000000	113.000	139.00000	TRUE
co2_total	3.200000	17.1000	21.100000	25.400	37.00000	TRUE
creatinina	0.100000	0.5900	1.020000	1.920	10.30000	TRUE
cto2	2.000000	11.5750	13.500000	16.125	25.70000	TRUE
dav_a	0.000000	207.0000	871.000000	2291.000	20280.00000	TRUE
dav_r	0.000000	2.0000	8.000000	19.000	94.00000	TRUE
eosinofilos_a	0.000000	0.0000	76.200000	197.650	4056.00000	FALSE
eosinofilos_r	0.000000	0.0000	1.000000	1.000	33.00000	FALSE
excesso_de_base	-27.500000	-8.7000	-3.600000	0.100	12.00000	TRUE
fosforo	1.500000	3.5000	4.100000	5.250	9.20000	TRUE
glicose	34.000000	95.5000	122.000000	174.000	438.00000	TRUE
globulina	1.300000	2.2000	2.600000	3.200	4.30000	TRUE
hcm	19.900000	27.7250	29.350000	31.100	41.10000	TRUE
hco3	0.000000	17.3000	21.500000	24.700	35.50000	TRUE
hemacias	1.280000	2.9600	3.540000	4.120	6.15000	TRUE
hemoglobina_p	4.600000	8.6000	10.300000	12.075	19.60000	TRUE
hemoglobina_reduzida	0.000000	1.8000	3.400000	6.200	78.40000	TRUE
idade	0.000000	4.0000	47.000000	67.500	94.00000	TRUE
kptt_seg	19.400000	30.9250	35.700000	43.100	247.50000	TRUE
lactato	0.400000	1.4000	2.100000	3.725	16.00000	TRUE
leucocitos	210.000000	7440.0000	12450.000000	18770.000	69230.00000	TRUE
linfocitos_a	39.900000	625.4500	1114.100000	2051.250	10119.90000	TRUE
linfocitos_atipicos_a	0.000000	0.0000	0.000000	0.000	1792.50000	FALSE
linfocitos_atipicos_r	0.000000	0.0000	0.000000	0.000	75.00000	FALSE
linfocitos_r	1.000000	5.0000	10.000000	19.750	90.00000	TRUE
magnesio	0.800000	1.6000	1.800000	2.100	4.00000	TRUE
meta_a	0.000000	0.0000	0.000000	106.750	3106.00000	FALSE

	0%	25%	50%	75%	100%	L
meta_r	0.000000	0.0000	0.000000	1.000	20.00000	FALSE
metahemoglobina	0.000000	0.7000	1.000000	1.300	3.50000	TRUE
mielocitos_a	0.000000	0.0000	0.000000	0.000	3286.00000	FALSE
mielocitos_r	0.000000	0.0000	0.000000	0.000	21.00000	FALSE
monocitos_a	0.000000	261.3000	591.000000	1102.200	4155.80000	TRUE
monocitos_r	0.000000	3.0000	5.000000	8.000	29.00000	TRUE
neutrofilos_a	4.000000	5245.0000	9864.000000	15595.000	44443.00000	TRUE
neutrofilos_r	1.000000	71.0000	83.000000	90.000	98.00000	TRUE
nlcr	0.011396	3.5304	8.099857	18.000	99.73743	TRUE
oxihemoglobina	19.600000	91.1000	94.300000	95.800	98.20000	TRUE
p50	18.600000	24.2000	26.060000	29.125	61.93000	TRUE
pco2	10.200000	30.1000	34.900000	42.400	171.60000	TRUE
pcr	0.500000	5.6000	17.300000	26.975	87.10000	TRUE
ph	6.720000	7.2900	7.380000	7.440	7.94000	TRUE
plaquetas	5000.000000	118000.0000	181000.000000	296000.000	865000.00000	TRUE
po2	24.300000	69.9000	87.200000	118.600	368.80000	TRUE
potassio	1.400000	3.4000	3.850000	4.500	7.20000	TRUE
promielocitos_a	0.000000	0.0000	0.000000	0.000	1970.00000	FALSE
promielocitos_r	0.000000	0.0000	0.000000	0.000	2.00000	FALSE
prot_totais	2.700000	4.1750	4.850000	5.500	6.50000	TRUE
rdw	12.000000	14.2000	15.500000	17.200	25.60000	TRUE
saturacao	20.000000	93.6000	96.500000	98.200	100.00000	TRUE
segmentados_a	4.500000	4535.0000	8124.300000	13447.200	45083.60000	TRUE
segmentados_r	1.000000	54.0000	70.000000	80.000	96.00000	TRUE
sodio	115.000000	133.0000	136.000000	140.000	171.00000	TRUE
tap	10.000000	46.0500	61.200000	77.900	100.00000	TRUE
tap_rni	1.000000	1.1600	1.350000	1.630	8.07000	TRUE
ureia	8.000000	36.0000	59.500000	109.000	347.00000	TRUE
vcm	62.500000	83.7250	88.100000	93.150	119.50000	TRUE
vg	12.900000	26.4250	31.100000	36.075	55.90000	TRUE

Only variables with different quartiles are kept. The variables **basofilos_a, basofilos_r, blastos_a, blastos_r, eosinofilos_a, eosinofilos_r, linfocitos_atipicos_a, linfocitos_atipicos_r, meta_a, meta_r, mielocitos_a, mielocitos_r, promielocitos_a, promielocitos_r** have been removed.

Hide

```
X       <- X[, -which(qtl$L == FALSE)]
drop    <- apply(X, 1, function(x) sum(is.na(x)) >= 0.6 * length(x))
X       <- X[!drop,]
gram    <- gram[!drop]
sexo    <- sexo[!drop]
```

Pearson correlation coefficient

Hide

```
PCOR    <-  0.80
dropx   <-  caret::findCorrelation(cor(X, use = "pairwise.complete.obs"), cutoff = PCOR, exact = T)

X1      <-  X[,-dropx]
```

Highly correlated variables were identified and excluded. As a cut-off point, Pearson's Correlation Coefficient of **0.8** was adopted in absolute value. Removed **fosforo, hco3, excesso_de_base, neutrofilos_r, dav_r, dav_a, neutrofilos_a, globulina, leucocitos, oxihemoglobina, saturacao, cto2, hemoglobina_p, vg, ast_tgo, vcm** variables. In the database you now have **424** observations and **39** variables. It is noteworthy that individuals with 60% or more of variables with missing information were eliminated.

Percentage of Missing Values and/or Zeros per Variable

Hide

```
PMISS   <-  30
pz        <-    apply(X1, 2, pctmisszeros)
X2        <-    X1[, -which(pz >= PMISS)]
dados0  <-  data.frame(X2, gram)
```

Note 1: Removing variables with **30%** or more missing values now has **31** variables and **424* * comments. It is worth remembering that there is still the presence of lost values. The percentages in these remaining variables are:

Hide

```
apply(X2, 2, pctmiss)
```

```
       bastonetes_a         bastonetes_r      calcio_ionizado   carboxihemoglobina                 chcm
cloreto            co2_total           creatinina              glicose
          0.4716981            0.4716981           12.2641509           22.4056604            0.4716981
12.2641509           22.4056604           14.8584906           18.1603774
                hcm             hemacias hemoglobina_reduzida                idade              lactato
linfocitos_a         linfocitos_r      metahemoglobina          monocitos_a
          0.4716981            0.7075472           22.4056604            0.0000000           22.6415094
0.4716981            0.4716981           22.8773585            0.7075472
        monocitos_r                 nlcr                  p50                 pco2                  pcr
ph            plaquetas                  po2             potassio
          0.4716981            0.4716981           22.6415094           22.4056604           17.6886792
22.4056604            0.7075472           22.4056604           12.2641509
                rdw        segmentados_a        segmentados_r                sodio
          0.7075472            0.7075472            0.4716981           12.2641509
```

Note 2: From this scenario onwards, several filtering criteria can be adopted before formulating a prediction model.

Strategy 1: Consider the database with **31** variables and **424** observations and do the imputation in the missing values (there are an infinite number of criteria for imputation and there is no consensus on the best criterion). A positive point of imputation is to keep the number of observations and the number of variables. It is important to note that keeping the **31** variables and removing the missing values gives **424** observations **(all values mentioned here are point dependent cutoff adopted above)**.

Strategy 2: According to some statistical criterion, exclude non-significant variables. For example, the Kolmogorov-Smirnov test, the t-Student and the Wilcoxon-Mann-Whitney test. Regardless of the test adopted, a cut-off point (for example, a 5% significance level) must be established for the exclusion and **(1)** impute the missing values of the remaining variables or **(2)** remove cases with at least one missing observation.

In this work, variables with value.p ≤ 0.1 were kept in at least one of the tests. The variables kept are **bastonetes_r, calcio_ionizado, chcm, co2_total, creatinina, hcm, hemacias, idade, lactato, monocitos_a, monocitos_r, p50, plaquetas, bastonetes_a, metahemoglobina, ph, gram**. For these variables, cases with at least one missed observation were removed. That left **320** observations and **16** variables (**0** cases negative and **0** positive cases). Again, the number of observations/variables remaining depends on the cut-off points adopted in all previous steps. Also, the number of observations/variables remaining depends on the order of filtering operations.

Two-Sample Kolmogorov–Smirnov Test, Two-Sample t-Test and Two Sample Wilcoxon Signed Rank Test

Hide

```
L       <- ncol(X2)
ks      <- lapply(1:L, function(i) ks.test(X2[gram =='positivo', i], X2[gram =='negativo', i])$p.value)
ts      <- lapply(1:L, function(i) t.test(X2[gram =='positivo', i], X2[gram =='negativo', i])$p.value)
wm      <- lapply(1:L, function(i) wilcox.test(X2[gram =='positivo', i], X2[gram =='negativo', i])$p.va
lue)
testes  <- data.frame(KS = unlist(ks), Tstudent = unlist(ts), WMW = unlist(wm))
rownames(testes) <- names(X2)
kable(testes, digits = 4)
```

	KS	Tstudent	WMW
bastonetes_a	0.3652	0.0717	0.1613
bastonetes_r	0.0376	0.0339	0.0204
calcio_ionizado	0.0406	0.0040	0.0105
carboxihemoglobina	0.7841	0.9296	0.6171
chcm	0.0171	0.1598	0.0689
cloreto	0.9713	0.7640	0.8663
co2_total	0.0435	0.0208	0.0385
creatinina	0.0005	0.0001	0.0000
glicose	0.6685	0.2010	0.4797
hcm	0.0671	0.0202	0.0168
hemacias	0.0176	0.0230	0.0156
hemoglobina_reduzida	0.6853	0.3912	0.3218
idade	0.0127	0.0029	0.0022
lactato	0.0118	0.0011	0.0008
linfocitos_a	0.3952	0.1223	0.1544
linfocitos_r	0.9640	0.7192	0.6066
metahemoglobina	0.2798	0.0335	0.0858
monocitos_a	0.0293	0.0442	0.0134
monocitos_r	0.0071	0.0050	0.0007
nlcr	0.7634	0.9194	0.4294
p50	0.0043	0.0007	0.0034
pco2	0.9692	0.9525	0.6345
pcr	0.7071	0.5083	0.6188
ph	0.1432	0.0061	0.0575
plaquetas	0.0012	0.0340	0.0085
po2	0.8413	0.1428	0.7255
potassio	0.7942	0.4537	0.8097
rdw	0.5765	0.1438	0.2076
segmentados_a	0.4659	0.6161	0.3722
segmentados_r	0.3236	0.6001	0.6440
sodio	0.8996	0.8155	0.7542

Hide

```
PVALOR  <-  0.10
keep    <-  significativas(X = testes, pvalor = PVALOR)
X3      <-  X2[,keep]
X4      <-  rmperdidos(data.frame(X3, gram))
```

Note that in general the tests reject the null hypothesis for significance level **10%** although the hypotheses under judgment are different. It is worth mentioning that some works in the literature use these tests for pre-selection of variables. See for example, **"The Kolmogorov filter for variable screening in high-dimensional binary classification QING MAI, HUI ZOU Biometrika, Vol. 100, No. 1 (MARCH 2013), pp. 229-234"** and references apud. Naturally, the big obstacle we have here is the sparseness and amount of lost observations.

Descriptive Statistics Variables According to Gram Classification

Hide

```
Sx  <- X4 %>% dplyr::select(-gram)
Sy  <- X4 %>% dplyr::select(gram)
aux <- lapply(Sx, function(x) tapply(x, Sy, descrSingle))
L   <- length(aux)
aux <-  lapply(1:L, function(i) plyr::ldply(aux[[i]], .id = names(aux[i])))
kable(aux)
```

bastonetes_r	n.validos	media	dp	minimo	mediana	maximo
positivo	151	11.25166	12.60170	0	7	63
negativo	169	14.05325	13.24192	0	11	61

calcio_ionizado	n.validos	media	dp	minimo	mediana	maximo
positivo	151	4.762053	0.4374476	3.84	4.67	6.41
negativo	169	4.616509	0.4880479	3.08	4.63	6.77

chcm	n.validos	media	dp	minimo	mediana	maximo
positivo	151	33.46225	1.529651	27.5	33.6	36.3
negativo	169	33.15030	1.510484	28.8	33.1	36.2

co2_total	n.validos	media	dp	minimo	mediana	maximo
positivo	151	21.90066	6.306827	4.1	21.8	37.0
negativo	169	20.36805	6.505203	3.2	20.8	34.6

creatinina	n.validos	media	dp	minimo	mediana	maximo
positivo	151	1.192517	1.017683	0.1	0.90	5.62
negativo	169	1.900473	1.685814	0.1	1.29	10.30

hcm	n.validos	media	dp	minimo	mediana	maximo
positivo	151	29.81788	2.839744	20.9	29.5	41.1
negativo	169	29.35799	2.868042	19.9	29.1	40.6

hemacias	n.validos	media	dp	minimo	mediana	maximo
positivo	151	3.640066	0.7087026	1.97	3.62	6.15
negativo	169	3.427811	0.8905813	1.28	3.34	5.56

idade	n.validos	media	dp	minimo	mediana	maximo
positivo	151	40.04901	30.30195	0	46	90
negativo	169	50.06450	28.33049	0	59	94

lactato	n.validos	media	dp	minimo	mediana	maximo
positivo	151	2.595364	2.256556	0.4	1.8	12.8
negativo	169	3.501775	2.941927	0.6	2.4	16.0

monocitos_a	n.validos	media	dp	minimo	mediana	maximo
positivo	151	784.6457	686.4323	0	653.1	4155.8
negativo	169	741.9994	712.8789	0	528.0	3393.5

monocitos_r	n.validos	media	dp	minimo	mediana	maximo
positivo	151	5.860927	3.920952	0	5	21
negativo	169	5.171598	3.871464	0	4	21

p50	n.validos	media	dp	minimo	mediana	maximo

p50	n.validos	media	dp	minimo	mediana	maximo
positivo	151	26.45709	4.096606	18.89	25.70	42.92
negativo	169	28.28308	6.204030	18.60	26.51	61.93

plaquetas	n.validos	media	dp	minimo	mediana	maximo
positivo	151	228258.3	142514.6	9000	198000	865000
negativo	169	184929.0	132733.8	9000	147000	671000

bastonetes_a	n.validos	media	dp	minimo	mediana	maximo
positivo	151	1692.06	2463.456	0	939	17369
negativo	169	2197.97	3015.570	0	932	19953

metahemoglobina	n.validos	media	dp	minimo	mediana	maximo
positivo	151	0.992053	0.4549759	0.1	0.9	2.1
negativo	169	1.131953	0.6136709	0.0	1.1	3.5

ph	n.validos	media	dp	minimo	mediana	maximo
positivo	151	7.376225	0.1089327	7.00	7.39	7.72
negativo	169	7.334675	0.1671257	6.73	7.38	7.94

Hide

```
NA
```

Fit, Validation and Prediction — Considering Only Previously Filtered Variables

The data used as a test are the 2019 data. In this test dataset there is information about **68 patients**, where **0** tested negative and **0** tested positive.

Hide

```
    controlTRT <- trainControl(method = "repeatedcv", number = 10, repeats = 10, savePredictions = TRUE, re
turnResamp = "final", classProbs = TRUE, allowParallel = TRUE, summaryFunction = multiClassSummary)

    dados   <-  X4;
    Teste   <-  teste2019[, names(dados)]

    set.seed(123)
    full    <-  train(gram ~., data = dados, method = "glm", family = "binomial", trControl = controlTRT, m
etric = "Accuracy", preProc = c("center", "scale"))

    #Otimista
    predprob    <-  predict(full, dados, 'prob')
    predclass   <-  predict(full, dados)
    aux         <-  data.frame(pred = predclass, obs = dados$gram, positivo = predprob$positivo, negativo =
predprob$negativo)
    m1          <-  c(multiClassSummary(aux,lev=c("positivo", "negativo"))[c(2, 4, 7:10)], rep(NaN, 6))

    #Predicao
    predprob    <-  predict(full, Teste, 'prob')
    predclass   <-  predict(full, Teste)
    aux         <-  data.frame(obs = Teste$gram, pred = predclass, negativo = predprob$negativo, positivo =
predprob$positivo)
    m2          <-  c(multiClassSummary(aux,lev=c("negativo", "positivo"))[c(2, 4, 7:10)], rep(NaN, 6))

    m3          <-  round(full$results[c(3, 5, 8:11, 17, 19, 22:25)],4)
    nomes       <-  names(m3)
    m3          <-  data.frame(CV = matrix(m3, ncol = 1)); rownames(m3) = nomes
    R           <-  data.frame(m3, Treinamento = m1, Teste = m2)

    kable(R, digits = 4)
```

	CV	Treinamento	Teste
AUC	0.6513	0.7153	0.6720
Accuracy	0.6014	0.6500	0.6324
Sensitivity	0.553	0.6225	0.6207
Specificity	0.6445	0.6746	0.6410
Pos_Pred_Value	0.5856	0.6309	0.5625
Neg_Pred_Value	0.6206	0.6667	0.6944
AUCSD	0.0817	NaN	NaN
AccuracySD	0.0694	NaN	NaN
SensitivitySD	0.1185	NaN	NaN
SpecificitySD	0.107	NaN	NaN
Pos_Pred_ValueSD	0.0829	NaN	NaN
Neg_Pred_ValueSD	0.0722	NaN	NaN

Hide

```
    preProcValues   <-  preProcess(dados, method = c("center", "scale"))
    train           <-  predict(preProcValues, dados)
    test            <-  predict(preProcValues, Teste)

    #write.table(train, file = "/home/jmazucheli/Dropbox/daniela2020/modelos_finais_notebook_1/treinamento_
todos.csv", row.names = F, sep = ";")
    #write.table( test, file = "/home/jmazucheli/Dropbox/daniela2020/modelos_finais_notebook_1/teste_todos.
csv",       row.names = F, sep = ";")
```

The estimated coefficients of each variable are:

Hide

```
    summary(full)
```

```
Call:
NULL

Deviance Residuals: 
    Min       1Q   Median       3Q      Max  
-2.0852  -1.0800   0.5229   1.0066   1.8286  

Coefficients:
                  Estimate Std. Error z value Pr(>|z|)   
(Intercept)      0.1460525  0.1220808   1.196  0.23156   
bastonetes_r     0.0262196  0.2052992   0.128  0.89838   
calcio_ionizado -0.1845076  0.1499433  -1.231  0.21850   
chcm             0.0631238  0.1659371   0.380  0.70364   
co2_total        0.0997206  0.1675879   0.595  0.55182   
creatinina       0.3971299  0.1800054   2.206  0.02737 * 
hcm             -0.3677340  0.1574542  -2.335  0.01952 * 
hemacias        -0.3959749  0.1408807  -2.811  0.00494 **
idade            0.0758804  0.1528767   0.496  0.61965   
lactato          0.2682459  0.1665468   1.611  0.10726   
monocitos_a      0.0984147  0.1898843   0.518  0.60426   
monocitos_r     -0.0009101  0.1799829  -0.005  0.99597   
p50              0.0561124  0.1988369   0.282  0.77779   
plaquetas       -0.2776667  0.1390602  -1.997  0.04585 * 
bastonetes_a    -0.0993086  0.2202890  -0.451  0.65213   
metahemoglobina  0.1539890  0.1303760   1.181  0.23756   
ph              -0.1167602  0.2078578  -0.562  0.57430   
---
Signif. codes:  0 ‘***’ 0.001 ‘**’ 0.01 ‘*’ 0.05 ‘.’ 0.1 ‘ ’ 1

(Dispersion parameter for binomial family taken to be 1)

    Null deviance: 442.6  on 319  degrees of freedom
Residual deviance: 394.8  on 303  degrees of freedom
AIC: 428.8

Number of Fisher Scoring iterations: 4
```

Hide

```
significativas <- names(which(coef(summary(full))[,4] <= 0.05))
```

It is important to note that only the **creatinina, hcm, hemacias, plaquetas** variables are statistically significant at the 5% significance level. Considering only these variables, the following results are obtained:

Hide

```
    controlTRT <- trainControl(method = "repeatedcv", number = 10, repeats = 10, savePredictions = 'all', r
eturnResamp = "all", classProbs = TRUE, allowParallel = TRUE, summaryFunction = multiClassSummary)

    dados   <-  X4[, c("gram", significativas)] #%>% mutate_if(is.numeric, logzero);
    Teste   <-  teste2019[, c("gram", significativas)] #%>% mutate_if(is.numeric,logzero)

    set.seed(123)
    fullS   <-  train(gram ~., data = dados, method = "glm", family = "binomial", trControl = controlTRT, m
etric = "Accuracy", preProc = c("center", "scale"))

    #Otimista
    predprob    <-  predict(fullS, dados, 'prob')
    predclass   <-  predict(fullS, dados)
    aux         <-  data.frame(obs = dados$gram, pred = predclass, negativo = predprob$negativo, positivo =
predprob$positivo)
    m1          <-  c(multiClassSummary(aux,lev=c("negativo", "positivo"))[c(2, 4, 7:10)], rep(NaN, 6))

    #Predicao
    predprob    <-  predict(fullS, Teste, 'prob')
    predclass   <-  predict(fullS, Teste)
    aux         <-  data.frame(obs = teste$gram, pred = predclass, negativo = predprob$negativo, positivo =
predprob$positivo)
    m2          <-  c(multiClassSummary(aux,lev=c("negativo", "positivo"))[c(2, 4, 7:10)], rep(NaN, 6))

    m3          <-  round(fullS$results[c(3, 5, 8:11, 17, 19, 22:25)],4)
    nomes       <-  names(m3)
    m3          <-  data.frame(CV = matrix(m3, ncol = 1)); rownames(m3) = nomes
    R           <-  data.frame(m3, Treinamento = m1, Teste = m2)

    kable(R, digits = 4)
```

	CV	Treinamento	Teste
AUC	0.6733	0.6897	0.6844
Accuracy	0.6296	0.6406	0.6618
Sensitivity	0.6028	0.6093	0.5862
Specificity	0.6536	0.6686	0.7179
Pos_Pred_Value	0.6101	0.6216	0.6071
Neg_Pred_Value	0.6524	0.6570	0.7000
AUCSD	0.0844	NaN	NaN
AccuracySD	0.0667	NaN	NaN
SensitivitySD	0.1176	NaN	NaN
SpecificitySD	0.0956	NaN	NaN
Pos_Pred_ValueSD	0.0718	NaN	NaN
Neg_Pred_ValueSD	0.0711	NaN	NaN

Hide

```
    preProcValues   <-  preProcess(dados, method = c("center", "scale"))
    train           <-  predict(preProcValues, dados)
    test            <-  predict(preProcValues, Teste)

    #write.table(train, file = "/home/jmazucheli/Dropbox/daniela2020/modelos_finais_notebook_1/treinamento_
reduzido.csv", row.names = F, sep = ";")
    #write.table( test, file = "/home/jmazucheli/Dropbox/daniela2020/modelos_finais_notebook_1/teste_reduzi
do.csv",       row.names = F, sep = ";")
```

For the reduced model we have:

Hide

```
    summary(fullS)
```

```
Call:
NULL

Deviance Residuals:
    Min       1Q   Median       3Q      Max
-1.9636  -1.0915   0.5629   1.0509   1.8151

Coefficients:
            Estimate Std. Error z value Pr(>|z|)
(Intercept)   0.1426     0.1198   1.190 0.234103
creatinina    0.5283     0.1448   3.649 0.000264 ***
hcm          -0.3446     0.1304  -2.642 0.008240 **
hemacias     -0.3586     0.1259  -2.849 0.004393 **
plaquetas    -0.3523     0.1288  -2.735 0.006242 **
---
Signif. codes:  0 ‘***’ 0.001 ‘**’ 0.01 ‘*’ 0.05 ‘.’ 0.1 ‘ ’ 1

(Dispersion parameter for binomial family taken to be 1)

    Null deviance: 442.60  on 319  degrees of freedom
Residual deviance: 404.46  on 315  degrees of freedom
AIC: 414.46

Number of Fisher Scoring iterations: 4
```

Hide

```
anova(full$finalModel, fullS$finalModel, test="LR")
```

```
Analysis of Deviance Table

Model 1: .outcome ~ bastonetes_r + calcio_ionizado + chcm + co2_total +
    creatinina + hcm + hemacias + idade + lactato + monocitos_a +
    monocitos_r + p50 + plaquetas + bastonetes_a + metahemoglobina +
    ph
Model 2: .outcome ~ creatinina + hcm + hemacias + plaquetas
  Resid. Df Resid. Dev  Df Deviance Pr(>Chi)
1       303     394.80
2       315     404.46 -12  -9.6554   0.6462
```

By the likelihood ratio test, the reduced model is not statistically different from the full model.
