# Supplementary material for: Routine laboratory biomarkers used to predict Gram-positive or Gram-negative bacteria involved in bloodstream infections
Source: Sci Rep. 2022 Sep 14;12:15466. doi: 10.1038/s41598-022-19643-1 (PMC9474441; doi:10.1038/s41598-022-19643-1)
Supplement: Supplementary file 3 — Supplementary Table S1. [file 41598_2022_19643_MOESM3_ESM.docx]

**Table S1.** Univariate analysis of routine laboratory biomarkers collected together with blood culture.

| **Biomarkers** | **n** | **Gram-positive** | **n** | **Gram-negative** | ***p*-value** | ***AUC*** |
| --- | --- | --- | --- | --- | --- | --- |
| Age | 217 | 37 (0-90) | 238 | 45 (0-94) | <0.001 | 0.58 |
| ALAT (U/L) | 61 | 122 (12-1684) | 79 | 86 (21-828) | 0.29 | 0.53 |
| Albumin (G/dL) | 10 | 2.3 (1.9-2.8) | 30 | 2.8 (1.1-3.9) | 0.46 | 0.59 |
| aPTT (sec) | 56 | 39.9 (20.2-115.5) | 78 | 45.7 (19.4-247.5) | 0.025 | 0.59 |
| ASAT (U/L) | 62 | 92.9 (15-1055) | 78 | 256 (11- 8013) | 0.17 | 0.55 |
| base excess (mmol/L) | 163 | -3.5 (-23.7-12) | 174 | -5.9 (-27.5-9.6) | 0.001 | 0.57 |
| Left shift (/mm^3^) | 200 | 1657 (0-17369) | 222 | 2578 (0-92581) | 0.87 | 0.56 |
| Left shift (%) | 200 | 11 (0-65) | 222 | 14 (0-94) | 0.015 | 0.56 |
| Calcium ionic (mg/dL) | 182 | 4.8 (3.84-6.41) | 200 | 4.6 (3.08-6.77) | 0.004 | 0.58 |
| Carboxyhemoglobin (%) | 163 | 1.4 (0-4.5) | 174 | 1.4 (-0.2-2.6) | 0.80 | 0.43 |
| Chloride (mg/dL) | 182 | 108.5 (89-136) | 200 | 108.7 (86-139) | 0.79 | 0.46 |
| CO2 total (mmol/L) | 163 | 22.0 (4.1-37) | 174 | 20.4 (3.2-34.6) | 0.021 | 0.56 |
| Creatinine (mg/dl) | 177 | 1.2 (0.1-10.1) | 192 | 1.8 (0.1-10.3) | <0.001 | 0.63 |
| CRP (mg/dl) | 167 | 17.9 (0.5-57.3) | 183 | 18.8 (0.5-87.1) | 0.54 | 0.51 |
| ctO_2_ (mL/dL) | 163 | 14.6 (7-23) | 173 | 13.3 (2-25.7) | 0.001 | 0.61 |
| Eosinophils % | 200 | 1.9 (0-33) | 222 | 1.3 (0-20) | 0.028 | 0.56 |
| Eosinophils (/mm^3^) | 200 | 216 (0-3217) | 222 | 170.3 (0-4056) | 0.79 | 0.57 |
| Glucoses (mg/dl) | 172 | 147.9 (36-438) | 184 | 138.2 (11-428) | 0.22 | 0.52 |
| Haematocrit (%) | 200 | 32.5 (20.4-55.9) | 222 | 30.26 (12.9-55.6) | <0.001 | 0.59 |
| Haemoglobin (G/dL) | 200 | 10.8 (6.5-19.5) | 222 | 10.1 (4.6-19.6) | <0.001 | 0.59 |
| HCO_3_ (mmol/L) | 163 | 21.7 (8.2-35.5) | 174 | 19.9 (0-33.6) | 0.001 | 0.57 |
| Lactate (mmol/L) | 163 | 2.6 (0.4-12.8) | 173 | 3.5 (0.6-16) | <0.001 | 0.60 |
| Lymphocytes (%) | 200 | 14.9 (1-78) | 222 | 14.5 (1-90) | 0.71 | 0.47 |
| Lymphocytes (/mm^3^) | 200 | 1693 (39.9-9158) | 222 | 1474 (77.5-10119) | 0.12 | 0.54 |
| Magnesium (mg/dl) | 85 | 1.9 (1-4) | 101 | 1.8 (0.8-3.3) | 0.42 | 0.50 |
| MCH (fl) | 200 | 29.8 (24.4-39.9) | 222 | 29.1 (19.9-40.6) | 0.02 | 0.56 |
| MCHC (g/dl) | 200 | 33.4 (29.9-36.2) | 222 | 33.2 (28.8-36.2) | 0.15 | 0.55 |
| MCV (pg) | 200 | 89.2 (62.9-116.8) | 222 | 87.6 (62.5-119.5) | 0.05 | 0.54 |
| Metahaemoglobin (%) | 161 | 0.9 (0.1-2.1) | 174 | 1.1 (0-3.5) | 0.023 | 0.56 |
| Monocytes % | 200 | 6.3(0-24) | 222 | 5.2 (0-29) | 0.005 | 0.59 |
| Monocytes (/mm^3^) | 200 | 867(0-5997) | 222 | 707 (0-3393) | 0.025 | 0.57 |
| NLCR | 200 | 13.6 (0.11-99.73) | 222 | 13.8 (0.055-97.99) | 0.12 | 0.45 |
| Neutrophiles % | 200 | 76.5(1-97) | 222 | 78.4 (5-98) | 0.24 | 0.54 |
| Neutrophiles (/mm^3^) | 200 | 11502(4-44443) | 222 | 11956 (71-65768) | 0.61 | 0.46 |
| Oxihaemoglobin (%) | 163 | 92.7 (57.8-98.2) | 174 | 91.7 (19.6-98.2) | 0.21 | 0.55 |
| Oxygen Saturation (%) | 163 | 94.9 (59.8-100) | 174 | 94.1 (20-99.7) | 0.28 | 0.54 |
| pCO_2_ (mmHg) | 163 | 37.1 (10.2-103.7) | 174 | 37.6 (13.3-171.6) | 0.75 | 0.45 |
| pH | 163 | 7.37 (7-7.72) | 174 | 7.33 (6.72-7.94) | 0.003 | 0.55 |
| PLT (10^3^/mm^3^) | 199 | 234.6 (9-865) | 222 | 206.6 (5-780) | 0.03 | 0.57 |
| pO_2_ (mmHg) | 163 | 96.6 (32.8-348.3) | 174 | 104.2 (24.3-368.8) | 0.16 | 0.50 |
| Potassium (mmol/L) | 182 | 3.9 (1.4-6.7) | 200 | 4.0 (1.9-7.2) | 0.48 | 0.50 |
| *p*50 (mmHg) | 163 | 26.2 (18.89-42.92) | 173 | 28.4 (18.6-61.93) | <0.001 | 0.50 |
| RBC (millions/uL) | 200 | 3.7 (1.97-6.15) | 222 | 3.5 (1.28-5.56) | 0.02 | 0.56 |
| RDW (%) | 200 | 15.8 (12.4-25.6) | 222 | 16.3 (12-39) | 0.08 | 0.53 |
| Sodium (mmol/L) | 182 | 136.6 (117-157) | 200 | 136.4 (115-171) | 0.82 | 0.44 |
| TAP (%) | 63 | 67.3 (14.8-100) | 84 | 55.1 (10-100) | <0.001 | 0.65 |
| TAP- INR | 63 | 1.3 (1.0 - 5.47) | 84 | 1.7 (1.0 - 8.07) | 0.002 | 0.66 |
| TP (G/L) | 11 | 4.7 (2,7-6.5) | 29 | 4.8 (2.7-6.2) | 0.78 | 0.34 |
| Urea (mg/dl) | 96 | 68.3 (8-243) | 126 | 88.9 (10-347) | 0.008 | 0.59 |
| WBC (/mm^3^) | 200 | 14.6 (0.21-65) | 222 | 14.7 (0.25- 98.5) | 0.94 | 0.43 |

Data given as mean with minimum and maximum values, n = number of samples Student T Test, ALAT= alanine aminotransferase, aPTT = activated partial thromboplastin time, ASAT = aspartate aminotransferase, Left shift (bands+metamyelocyte+myelocyte+promyelocite+blasts), CO_2_= carbon dioxide; CRP = C-reactive protein, ctO_2_= oxygen contend, HCO_3_= sodium bicarbonate, MCH = mean corpuscular haemoglobin, MCV = mean corpuscular volume, MCHC = Mean corpuscular haemoglobin, NLCR= Neutrophiles/Lymphocytes, pCO_2_= carbon dioxide pressure, pH= hydrogen potential, PLT = platelet count, pO_2_= oxygen pressure, RBC = red blood cell count, RDW = red blood cell distribution width, TAP= prothrombin time, INR= international standardized ratio, TP = total protein, WBC = white blood cell count.
